# MicroRNA-570 is a novel regulator of cellular senescence and inflammaging

**DOI:** 10.1096/fj.201800965R

**Published:** 2018-08-29

**Authors:** Jonathan R. Baker, Chaitanya Vuppusetty, Thomas Colley, Shyreen Hassibi, Peter S. Fenwick, Louise E. Donnelly, Kazuhiro Ito, Peter J. Barnes

**Affiliations:** Airway Disease Section, National Heart and Lung Institute, Imperial College London, London, United Kingdom

**Keywords:** COPD, miRNA, cell cycle, epithelial cells, inflammation

## Abstract

Diseases of accelerated aging often occur together (multimorbidity), and their prevalence is increasing, with high societal and health care costs. Chronic obstructive pulmonary disease (COPD) is one such condition, in which one half of patients exhibit ≥4 age-related diseases. Diseases of accelerated aging share common molecular pathways, which lead to the detrimental accumulation of senescent cells. These senescent cells no longer divide but release multiple inflammatory proteins, known as the senescence-associated secretory phenotype, which may perpetuate and speed disease. Here, we show that inhibiting miR-570-3p, which is increased in COPD cells, reverses cellular senescence by restoring the antiaging molecule sirtuin-1. MiR-570-3p is induced by oxidative stress in airway epithelial cells through p38 MAP kinase-c-Jun signaling and drives senescence by inhibiting sirtuin-1. Inhibition of elevated miR-570-3p in COPD small airway epithelial cells, using an antagomir, restores sirtuin-1 and suppresses markers of cellular senescence (p16^INK4a^, p21^Waf1^, and p27^Kip1^), thereby restoring cellular growth by allowing progression through the cell cycle. MiR-570-3p inhibition also suppresses the senescence-associated secretory phenotype (matrix metalloproteinases-2/9, C-X-C motif chemokine ligand 8, IL-1β, and IL-6). Collectively, these data suggest that inhibiting miR-570-3p rejuvenates cells *via* restoration of sirtuin-1, reducing many of the abnormalities associated with cellular senescence.—Baker, J. R., Vuppusetty, C., Colley, T., Hassibi, S., Fenwick, P. S., Donnelly, L. E., Ito, K., Barnes, P. J. MicroRNA-570 is a novel regulator of cellular senescence and inflammaging.

Chronic obstructive pulmonary disease (COPD) is a global epidemic, with the disease predicted to be the third leading cause of death by 2020 (1, 2). COPD is characterized by chronic inflammation of the lung parenchyma and peripheral airways that is associated with destruction of alveolar walls (emphysema), fibrosis of small airways, and hypersecretion of mucus (chronic bronchitis). In addition to having affected lungs, patients with COPD have at least one associated chronic disease, with 50% of patients having ≥4 comorbidities (3). These multimorbidities may share common molecular pathways, which lead to chronic disease and are becoming increasingly prevalent as populations age (4, 5).

The major risk factors for COPD are exposure to chronic inhaled irritants, such as cigarette smoke, biomass smoke, and air pollution (6). These irritants release reactive oxygen species, which are also elevated in many age-related diseases (7) and may drive acceleration of the aging process *via* reactive oxygen species–induced cellular senescence (8). Patients with COPD are exposed to high levels of oxidative stress, both from inhaled irritants and chronic pulmonary inflammation (9). This exposure drives a senescent phenotype within the lungs and is believed to be an important driver of the pathophysiology of the disease (10). Currently, however, the mechanisms by which elevated levels of oxidative stress in COPD induce senescence remains unclear.

Cellular senescence can be defined as growth cycle arrest, generated *via* 2 distinct mechanisms: replicative and stress-induced senescence. Replicative senescence is believed to be a consequence of telomere shortening due to incomplete chromosomal replication during somatic cell divisions (11). In contrast, premature senescence is considered a response to many stress stimuli, including oxidative stress (12). Senescent cells are characterized by permanent cell cycle arrest induced by dysregulated p53-p21 and/or p16/Rb signaling. Senescent cells display altered cell morphology, becoming larger in size, flattened, and more vacuolized, as well up-regulating expression of β-galactosidase. Senescent cells also classically secrete a milieu of proinflammatory mediators known as the senescence-associated secretory phenotype (SASP) (13). The SASP includes proinflammatory cytokines, chemokines, growth factors, and matrix metalloproteinases (MMPs), all of which mirror the secretome of many chronic inflammatory diseases, including COPD (10).

To counteract oxidative stress and prevent accelerated aging, there are numerous endogenous antioxidant and antiaging molecules. However, in COPD and other age-related diseases, expression of several of these antioxidant genes is reduced, further increasing oxidative stress (7, 9). One of the most well-described antiaging proteins are sirtuins. These are class III protein deacetylases that catalyze NAD^+^-dependent deacetylation of target proteins (14). Sirtuin-1, a putative antiaging molecule, is the most well-characterized family member and has been implicated in the regulation of numerous biologic processes, including inflammation, cellular senescence, DNA repair, and life span. Sirtuin-1 deacetylates NF-κB, forkhead box class O (FOXO)-3, p21, p16, p53, Klotho, β-catenin/Wnt, and histones, all of which are associated with the cellular senescence and pathology of COPD (15–17). Expression of sirtuin-1 is reduced in the peripheral lungs of patients with COPD, and this finding can be mimicked *in vitro* by exogenous oxidative stress (17, 18).

Micro-RNAs (miRNAs) are small endogenous noncoding RNAs, typically 18–23 nt in length, that are involved in posttranscriptional regulation of gene expression. Mature miRNAs bind to target mRNAs at complementary sites within the 3′UTR, which can lead to decreased stability and translation of the target mRNA (19). Dysregulation of miRNAs in COPD has been well documented, but their functional roles have been less well studied. We have previously shown the importance of increased miR-34a in the down-regulation of sirtuin-1 in cell and tissue samples from patients with COPD, leading to the modulation of cell cycle checkpoint inhibitors and senescence (20). However, due to elevation of miR-34a in non–small cell lung cancer (21), there are concerns that miR-34a may not be a viable therapeutic target in COPD, as these patients have an increased risk of developing lung cancer (22). We therefore sought alternate miRNAs that may regulate sirtuin-1 and cellular senescence.

Utilizing miRNA target prediction sites, TargetScan (23) and MiRanda (24), we examined multiple miRNAs that may modulate sirtuin-1 and identified miR-570-3p (which was shown to have 3 putative binding sites) (Supplemental Fig. S1) as a potential novel regulator. MiR-570-3p has been previously described in asthma (25), with conflicting roles in regulating cellular growth (26, 27), but it has not been studied in the context of senescence, sirtuin-1, or COPD. Here, for the first time we report that miR-570-3p regulates cellular senescence and the SASP by inhibiting sirtuin-1. We show that inhibition of miR-570-3p by using an antagomir leads to the reversal of cellular senescence, with improved cellular growth and reduced expression of SASP inflammatory proteins, suggesting cellular rejuvenation.

## MATERIALS AND METHODS

Hydrogen peroxide (H_2_O_2_) was purchased from MilliporeSigma (Poole, United Kingdom). The p38 MAPK inhibitor VX745 was purchased from Tocris Bioscience (Bristol, United Kingdom) and the PI3K inhibitors: PIK75 hydrochloride (PI3Kα) from Abcam (Cambridge, United Kingdom) and GSK2636771 (PI3Kβ), AS-605240 (PI3Kγ), and IC-87114 (PI3Kδ) from VWR International (Lutterworth, United Kingdom). Antibodies against the following were used for immunoblotting: β-actin (AC-15, ab6276; Abcam) and sirtuin-1 (1F3; 8469; Cell Signaling Technology, Cambridge, United Kingdom). Anti-rabbit (P0448) and anti-mouse (P0260) secondary antibodies were from Agilent (Cambridge, United Kingdom), and Lipofectamine RNAiMAX and Lipofectamine LTX Plus were purchased from Thermo Fisher Scientific (Waltham, MA, USA).

### Lung tissue

COPD severity was assessed according to the Global Initiative for Chronic Obstructive Lung Disease classification based on spirometry results (28). Lung tissues were obtained from an established tissue bank linked to an established patient registry that has been used previously (29). Subjects were matched for age and normal smokers and COPD patients for smoking history (**Table 1**). mRNA and miRNAs were extracted as previously described (20).

**TABLE 1:** The characteristics of study subjects for lung homogenate samples

Characteristic	Nonsmokers (*n* = 9)	Smokers (*n* = 9)	GOLD Stage 1 (*n* = 8)	GOLD Stage 2 (*n* = 9)	GOLD Stage 3 (*n* = 4)	GOLD Stage 4 (*n* = 9)
Age (yr)	63.7 ± 13.7	63.7 ± 12.4	67.7 ± 7.0	63.0 ± 9.3	63.3 ± 9.1	59.8 ± 4.5
Sex (M:F)	2:7	3:6	5:3	4:5	3:1	3:6
FEV_1_ (L)	2.56 ± 0.6	2.74 ± 0.7	2.7 ± 0.6	1.8 ± 0.4*	1.7 ± 0.8*	0.5 ± 0.1***
FEV_1_ (% predicted)	97.2 ± 16.4	100.5 ± 15.3	89.1 ± 3.9	65.4 ± 17.5***	48.8 ± 21.4**	17.6 ± 3.5***
FVC (L)	3.2 ± 1.1	3.4 ± 0.9	4.0 ± 0.9	3.1 ± 0.8	3.6 ± 1.0	2.0 ± 0.5*
FEV_1_:FVC	80.3 ± 4.9	75.2 ± 4.53	64.3 ± 3.6*	61.5 ± 7.9**	49.5 ± 24.2**	27.0 ± 6.8***
Pack-years*^a^*	0 ± 0	45.9 ± 32.9	44.3 ± 17.0	57.7 ± 35.4	46.7 ± 21.8	38.5 ± 16.2

Data are expressed as means ± sd. Patients with COPD were categorized according to Global Initiative for Chronic Obstructive Lung Disease (GOLD) severity. F, female; FEV_1_, forced expiratory volume in 1 s; FVC, forced vital capacity; M, male. *^a^*Number of cigarettes smoked per day/20 × duration of smoking. *P < 0.05, **P < 0.01, ***P < 0.001, ^#^*P* < 0.05, ^##^*P* < 0.01 *vs.* nonsmoker.

### Cell culture and transfections

BEAS-2B cells (ATCC, Teddington, United Kingdom) and human primary small airway epithelial cells (SAECs) were cultured as previously described (20). The subjects were matched for age and smoking history (**Table 2**). Subjects provided informed consent, and the study was approved by the NRES London-Chelsea Research Ethics committee (study 09/H0801/85). Cells were serum-starved 16 h before stimulation. BEAS-2B and primary SAECs were transfected with siRNA or mirVana miRNA mimics and inhibitors by using Lipofectamine RNAiMAX for 24 h before stimulation with H_2_O_2_, mirVana miRNA Mimic Negative Control #1, has-miR-570-3p MC12799, has-miR-34a MC11030, and inhibitors mirVana miRNA Inhibitor Negative Control 1, and has-miR-570-3p MH12799 (30 or 60 nM) (Ambion, Foster City, CA, USA) as previously described. BEAS-2B cells were transfected with siRNA (c-Jun 6203 or PI3Kα 6359; Cell Signaling Technology), Negative Control 1, or SIRT1 (136457) (Ambion Silencer Select siRNA, 100 nM).

**TABLE 2:** The characteristics of study subjects for primary epithelial cells

Characteristic	Nonsmoker (*n* = 10)	COPD (*n* = 14)
Age (yr)	61.2 ± 18.4	66.75 ± 10.78
Sex (M:F)	02:08	07:07
FEV_1_ (L)	2.59 ± 1.08	1.32 ± /−0.65
FEV_1_ (% predicted)	91.71 ± 11.12	57.65 ± 28.55
FVC (L)	3.21 ± 1.02	2.76 ± 1.10
FEV_1_:FVC	0.78 ± 0.11	0.47 ± 0.19
Pack-years*^a^*	0	36.67 ± 18.56

Data are expressed as means ± sd. Patients with COPD were categorized according to Global Initiative for Chronic Obstructive Lung Disease definitions. *^a^*Number of cigarettes smoked per day/20 × duration of smoking. F, female; FEV_1_, forced expiratory volume in 1 s; FVC, forced vital capacity; M, male.

### RNA extraction and real-time quantitative PCR

mRNA and miRNAs were extracted by using the miRNeasy kit (Qiagen, Germantown, MD, USA) according to the manufacturer’s instructions. RNAs were then reverse-transcribed by using the TaqMan normal RNA and MicroRNA Reverse Transcription Kit (Thermo Fisher Scientific). Both normal and miRNA levels were detected according to the TaqMan Assays. After the reactions, the threshold cycle values were determined by using fixed-threshold settings. The following TaqMan assays were used: SIRT1 Hs01009006, hTERT Hs00972656, p21^Waf1^ Hs00355782, p16 ^INK4a^ Hs00923894, p27^Kip1^ Hs01597988, CDK4 Hs00364847, MMP9 Hs00234579, MMP2 Hs01548727, Jun Hs 01103882, SOD2 Hs00167309, FOXO3a Hs0818121, CXCL8 Hs00174103; or TaqMan MicroRNA Assay (hsa-miR-570-3p TM0002347) and TaqMan pri-MicroRNA assay (hsa-mir-570 Hs03304353) (Thermo Fisher Scientific). RNU48 (001006) was used as the control for miRNAs and guanine nucleotide binding protein-polypeptide 2-like 1 as the control for normal cDNA.

### Sputum samples

Sputum was induced by using 3% (weight/volume) nebulized hypertonic saline. Saliva was removed from the sputum samples and extracted as previously described (30); protein and RNA were extracted by using the mirVana PARIS RNA and Native Protein Purification Kit. RNA samples were reverse transcribed as previously stated and qPCR performed. **Table 3** presents patient demographic characteristics. Sputum samples were obtained by Dr. Andriana I. Papaioannou (Athens, Greece), and written informed consent was acquired.

**TABLE 3:** The characteristics of study subjects for sputum samples

Characteristic	Nonsmoker (*n* = 1)	Smokers without COPD (*n* = 4)	COPD1 (*n* = 2)	COPD2 (*n* = 3)	COPD3 (*n* = 6)	COPD4 (*n* = 2)
Age (yr)	65	65.5 ± 8.2	56 ± 7.1	72.0 ± 2.8	69.7 ± 6.3	70.5 ± 12.0
Sex (M:F)	1 (1/0)	4 (3/1)	2 (2/0)	3 (3/0)	6 (4/2)	2 (2/0)
FEV_1_ (L)	2.3	2.59 ± 0.72	3.42 ± 0.2	1.71 ± 0.24	0.97 ± 0.25	0.73 ± 0.07
FEV_1_ (% predicted)	92	9.25 ± 23.6	92.5 ± 4.95	58.7 ± 6.6	35.9 ± 3.9	23.5 ± 2.1
FEV_1_: FVC	77	82.3 ± 1.8	70 ± 4.3	45.0 ± 11.3	37.4 ± 6.8	31 ± 1.41
Pack-years*^a^*	0	21.5 ± 5.4	50	101.66 ± 85.48	60.33 ± 40.62	102.5 ± 45.96

Data are expressed as means ± sd. Patients with COPD were categorized according to Global Initiative for Chronic Obstructive Lung Disease definitions of severity. *^a^*Number of cigarettes smoked per day/20 × duration of smoking. F, female; FEV_1_, forced expiratory volume in 1 s; FVC, forced vital capacity; M, male.

### Luciferase assay

Luciferase experiments were performed as previously described using the Dual-Luciferase Reporter Assay System (Promega, Madison, WI, USA) (20). Briefly, BEAS-2B were seeded onto 24-well plates overnight. Cells were then transfected with 0.2 μg of Luc-SIRT1 3′UTR (Luc-SIRT1 3′UTR was a gift from Charles Lowenstein; plasmid 20379; Addgene, Cambridge, MA, USA) and 0.1 μg of Renilla expression vector using Lipofectamine LTX Plus reagent. These were cotransfected with 30 nM of mirVana mimics or control for 48 h. The dual-luciferase assay was conducted by using the Dual-Luciferase Reporter Assay System (Promega), with changes in firefly luciferase expression being normalized to Renilla expression.

### Western blotting

Protein extracts were prepared by using RIPA buffer (150 mM NaCl, 1.0% IGEPAL CA-630, 0.5% sodium deoxycholate, 0.1% SDS, and 50 mM Tris, pH 8.0.; MilliporeSigma) completed with protease inhibitor cocktail (Roche, Welwyn Garden City, United Kingdom). Protein extracts (40 µg) were analyzed by using SDS-PAGE (Thermo Fisher Scientific) and detected with Western blot analysis by chemiluminescence (ECL Plus; GE Healthcare, Hatfield, United Kingdom). Protein expression levels were expressed relative to β-actin.

### SA-β-galactosidase staining

Passage 2–3 SAECs from patients with COPD and nonsmokers were plated into 24-well plates and left for 24 h to adhere. Cells were then fixed, and senescence-associated β-galactosidase activity was determined according to the manufacturer’s instructions (ab65351; Abcam), and cells stained blue were counted as a proportion of total cells.

### iCELLigence cellular growth assay

For continuous monitoring of changes in cell growth, SAECs were seeded onto E-plates and incubated overnight, and ran on a RealTime Cell Analyzer station (iCELLigence System; Roche, Mannheim, Germany). Cells were then cultured with either miR-570-3p inhibitors or control inhibitors for 48 h; impedance was measured every hour for 48 h.

### Zymography

MMP2/9 enzyme activity was measured by zymography using Novex Zymogram Gelatin Gels (Thermo Fisher Scientific). Supernatant was diluted in Novex Tris-Glycine SDS sample buffer (Thermo Fisher Scientific) and ran on zymogram gel. After electrophoresis, gels were incubated with Novex zymogram renaturing buffer (Thermo Fisher Scientific) and gels incubated in Novex zymogram developing buffer (Thermo Fisher Scientific) for 18 h at 37°C. After incubation, gels were stained with a Colloidal Blue Staining Kit (Thermo Fisher Scientific).

### ELISA

C-X-C motif chemokine ligand 8 (CXCL8), IL-1β, TNF, CCL2, and IL-6 were quantified by using commercially available ELISA kits (R&D Systems Europe, Abingdon, United Kingdom) according to the manufacturer’s instructions.

### Statistical analysis

Data are expressed as means ± sem. Results were analyzed by using Mann-Whitney tests, paired or nonpaired Student’s *t* tests, and 1- or 2-way ANOVA for repeated measures with Dunnett or Bonferroni posttests. GraphPad Prism 6 software (GraphPad Software, La Jolla, CA, USA) was used for analyses. Clinical data were analyzed by using Kruskal-Wallis tests followed by Mann- Whitney tests. Correlation coefficients were calculated by using Spearman’s rank method. Values of *P* ≤ 0.05 were considered statistically significant.

## RESULTS

### MiR-570-3p directly binds sirtuin-1 and modulates its expression

**Figure 1:**
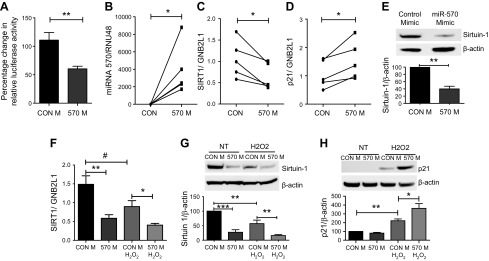
MiRNA-570 directly binds the 3′UTR of sirtuin-1 and is elevated in patients with COPD. *A*) The dual-luciferase reporter assays using vectors encoding sirtuin-1 target site in the 3′-UTR or control in BEAS-2B cells cotransfected with either an miR-570-3p mimic or mimic control. SAECs from nonsmokers (*n* = 5) were treated with miR-570-3p mimic or mimic control (CON) for 48 h, miRNA and RNA were extracted, and changes in gene expression assessed by qRT-PCR normalized to guanine nucleotide binding protein-polypeptide 2-like 1 (GNB2L1) or RNU48. *B–D*) Differences in the gene expression of miRNA-570-3p (*B*), sirtuin-1 (*C*), and p21 (*D*). *E*) Changes in the protein expression of sirtuin-1 after overexpression of miR-570-3p mimic. BEAS-2B cells were transfected with either an miR-570-3p mimic or mimic control (CON) and treated with or without H_2_O_2_ and mRNA or protein extracted. *F*–*H*) Sirtuin-1 gene expression (*F*) was assessed, as well as sirtuin-1 (*G*) and p21 protein (*H*) (*n* = 5). Data are means ± sem and were analyzed by using the Mann-Whitney *U* test, paired or unpaired Student’s *t* test, or Kruskal-Wallis test with *post hoc* Dunn’s test; *P < 0.05, **P < 0.01, ***P < 0.001, ^#^*P* < 0.05.

To assess whether sirtuin-1 is a direct target of miR-570-3p, a 3′UTR sirtuin-1 reporter construct was used to assess direct binding of miR-570-3p to sirtuin-1 mRNA. Overexpression of an miR-570-3p mimic compared with control significantly decreased luciferase reporter activity, suggesting miR-570-3p binds directly to sirtuin-1 mRNA (**Fig. 1*A***); a further reduction in reporter activity was seen when miR-570-3p was combined with a known sirtuin regulator, miR-34a (Supplemental Fig. S2). Overexpressing an miR-570-3p mimic in primary SAECs from nonsmokers (Fig. 1*B*) significantly reduced sirtuin-1 mRNA and protein expression, while elevating p21^Waf1^ mRNA (Fig. 1*C–E*). There was a concomitant increase in p16^INK4a^, p27^Kip1^, MMP-2, and MMP-9 expression, with reduced expression of cyclin-dependent kinase 4 (CDK4), as well as FOXO3a and superoxide dismutase 2 (SOD2), all of which are regulated by sirtuin-1 (Supplemental Fig. S3). In the bronchial epithelial cell line BEAS-2B, we confirmed that sirtuin-1 was reduced by the overexpression of the miR-570-3p mimic, in both the presence and absence of oxidative stress, compared with control (Fig. 1*F, G*). Overexpression of the mimic also significantly increased the protein expression of p21 in the presence of oxidative stress (Fig. 1*H*).

### MiR-570-3p and senescence markers are elevated in the lungs of patients with COPD

**Figure 2:**
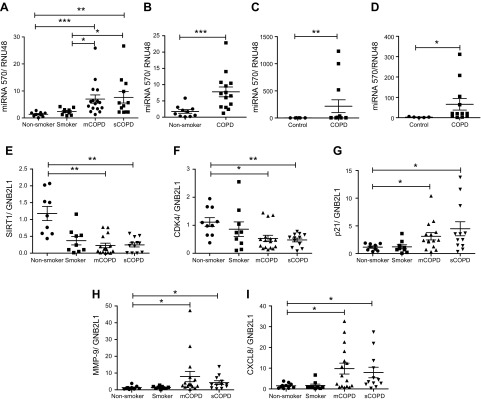
MiR-570-3p and senescence markers are elevated in lung tissue and cells from patients with COPD. *A*) Lung tissue from resections obtained from nonsmoker (*n* = 9) and non-COPD smokers (*n* = 9), moderate/mild COPD (mCOPD) (*n* = 16), and severe COPD (sCOPD) (*n* = 12), and RNA extracted and miR-570-3p expression detected. *B*) MiR-570-3p levels detected in SAECs from nonsmokers (*n* = 10) and patients with COPD (*n* = 14). *C*) MiR-570-3p levels in peripheral blood mononuclear cells from control [*n* = 10 (6 nonsmokers and 4 smokers)] and patients with COPD (*n* = 14). *D*) MiR-570-3p levels in induced sputum cells from control [*n* = 5 (1 nonsmoker and 4 smokers)] and patients with COPD (*n* = 12). *E–I*) Changes in the gene expression in lung homogenate samples of sirtuin-1 (*E*), CDK4 (*F*), p21^Waf1^ (*G*), MMP-9 (*H*), and CXCL8 (*I*). Data are means ± sem and were analyzed by using a Mann-Whitney *U* test, paired or unpaired Student’s *t* test, or Kruskal-Wallis test with *post hoc* Dunn’s test; *P < 0.05, **P < 0.01, ***P < 0.001.

Because miR-570-3p directly regulates sirtuin-1, it was important to determine whether expression of this miRNA is altered in disease*. MiR-570*-3p expression was significantly elevated in lung tissue homogenates from patients with both mild/moderate and severe COPD compared with age-matched nonsmokers and smokers without COPD (**Fig. 2*A***). Up-regulation of miR-570-3p correlated with decreased lung function, measured by forced expiratory volume in 1 s (% predicted), and the ratio of forced expiratory volume in 1 s to forced vital capacity, a measurement of airway obstruction (Supplemental Fig. S4). SAECs, which line the lumen of peripheral airways and are believed to be involved in early disease (31, 32), also exhibited increased expression of miRNA-570-3p (Fig. 2*B*). In addition to an increase in mature miR-570, there was elevated expression of the primary miRNA transcript in COPD cells compared with nonsmokers (Supplemental Fig. S5). To determine whether increased miR-570-3p was seen in other cells, miR-570-3p expression was examined in sputum and peripheral blood mononuclear cell samples from patients with COPD and again showed significant elevation compared with samples from age-matched controls (Fig. 2*C, D*). In addition to increased expression of miR-570-3p, lungs from patients with COPD also expressed increased markers of cellular senescence, with reduced gene expression of sirtuin-1 and CDK4 along with elevated p21 and the SASP proteins MMP-9 and CXCL8 (Fig. 2*E–I*).

### SAECs from patients with COPD display a senescence phenotype

**Figure 3:**
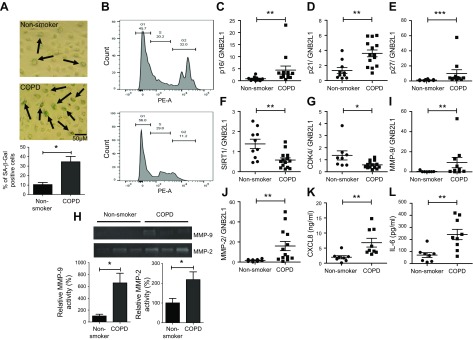
SAECs from patients with COPD display a cellular senescence phenotype. *A*) SAECs from nonsmokers and patients with COPD were stained for senescence-associated β-galactosidase (SA-β-Gal), and SA-β-Gal–positive counted (*n* = 4). *B*) Untreated passage 2–3 SAECs from nonsmokers and patients with COPD were stained with propidium iodide and fluorescence detected by using flow cytometry on the PE-A channel (*n* = 4). *C–G*) p16^INK4a^ (*C*), p21^Waf1^ (*D*), p27^Kip1^ (*E*), SIRT1 (*F*), and CKD4 (*G*) gene expression detected in SAECs from nonsmokers (*n* = 8–10) and patients with COPD (*n* = 11–14). *H*) Changes in MMP-9 and MMP-2 release from nonsmoker (*n* = 5) and COPD (*n* = 5) SAECs were detected by zymography. *I*, *J*) MMP-9 (*I*) and MMP-2 (*J*) gene expression were also detected in nonsmoker (*n* = 8–10) and COPD (*n* = 11–14) SAECs. *K*, *L*) Baseline release of CXCL8 (*K*) and IL-6 (*L*) from nonsmoker (*n* = 7) and COPD (*n* = 8) SAECs, as measured by using ELISA. Data are means ± sem and were analyzed by using a Mann-Whitney *U* test unpaired Student’s *t* test, or Kruskal-Wallis test with *post hoc* Dunn’s test; *P < 0.05, **P < 0.01, ***P < 0.001.

COPD SAECs displayed evidence of cellular senescence, with increased staining of senescence-associated β-galactosidase (34.5%) compared with age- and passage-matched SAECs from nonsmokers (10.5%) (**Fig. 3*A***). Flow cytometric analysis suggested COPD SAECs were mainly in G1 arrest (Fig. 3*B*), with elevated levels of the cell cycle checkpoint inhibitors (p16^INK4a^, p21^Waf1^, and p27^Kip1^) (Fig. 3*C–E*), and reduced expression of sirtuin-1 and CDK4 (Fig. 3*F, G*) compared with cells from age-matched nonsmokers. MMP-2 and MMP-9, components of the SASP, were elevated at both the mRNA and protein level in COPD SAECs (Fig. 3*H–J*). There was also a significant increase in the secretion of proinflammatory cytokines CXCL8 and IL-6 (Fig. 3*K, L*). Together, these data suggest that COPD SAECs display a senescence phenotype, with cell cycle arrest and increased release of proinflammatory mediators.

### MiR-570-3p is induced by oxidative stress *via* p38 MAP kinase-c-Jun signaling

**Figure 4:**
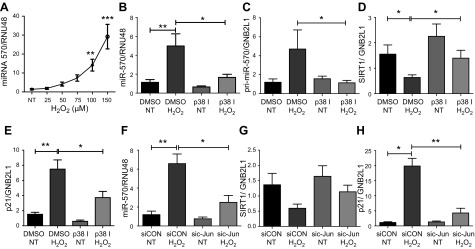
Oxidative stress induces miRNA-570-3p expression in a p38-c-jun–dependent manner. *A*) MiR-570-3p expression in BEAS-2B cells after H_2_O_2_ treatment (*n* = 5). *B–E*) p38MAPK inhibitor VX745 (100 nM) effect on miR-570-3p (*B*), pri-miR-570-3p (*C*), sirtuin-1 (*D*), and p21^Waf1^ (*E*) gene expression in BEAS-2B cells treated with or without H_2_O_2_ (*n* = 6). *F–H*) MiR-570-3p (*F*), sirtuin-1 (*G*), and p21^Waf1^ (*H*) gene expression in BEAS-2B cells were transfected with c-Jun siRNA (100 nM) or random oligonucleotide and then treated with or without H_2_O_2_ (*n* = 6). Data are means ± sem and were analyzed by using Kruskal-Wallis test with *post hoc* Dunn’s test, 1-way ANOVA with *post hoc* Bonferroni correction, unpaired or paired Student’s *t* test, and Wilcoxon signed rank test; *P < 0.05, **P < 0.01, ***P < 0.001.

Because oxidative stress induces cellular senescence (33), we investigated whether oxidative stress induced miR-570-3p expression. Treatment of BEAS-2B cells with increasing concentrations of H_2_O_2_ significantly increased miR-570-3p (**Fig. 4*A***). Our previous data showed that oxidative stress induced a well-recognized senescence-modulating miRNA, miR-34a, in a PI3Kα-dependent manner (20). However, PI3Kα inhibition, using either the pharmacologic inhibitor PIK75 or knock down with PI3Kα siRNA, failed to reduce miR-570-3p expression (Supplemental Fig. S6*A*, *D*); pharmacologic inhibition of PI3Kγ and PI3Kδ isoenzymes was also without effect (Supplemental Fig. S6*B*, *C*). Oxidative stress also activates the p38 MAPK pathway (34), which may modulate p21^Waf1^ and p16^INK4a^ expression (35, 36), inducing cellular senescence (37). Inhibition of p38MAPK, using the selective inhibitor VX745, significantly decreased oxidative stress–induced miR-570-3p and pri-miR-570-3p expression (Fig. 4*B, C*), with a concomitant rescue of sirtuin-1 and a significant decrease in p21^Waf1^ gene expression (Fig. 4*D, E*). To further understand this mechanism, c-Jun, a member of the *cis*-element of the transcription factor AP-1 that is modulated by p38MAPK (38), was knocked down by using siRNA. In c-Jun knock-down cells (Supplemental Fig. S7), miR-570-3p expression was significantly reduced (Fig. 4*F*), whereas sirtuin-1 was again rescued and p21^Waf1^ decreased (Fig. 4*G, H*). These data imply that oxidative stress induces the expression of miR-570-3p by activating p38MAPK, leading to AP-1–mediated transcription of miR-570-3p.

### MiR-570-3p antagomir rescues sirtuin-1 expression and suppresses cellular senescence markers in COPD SAECs

**Figure 5:**
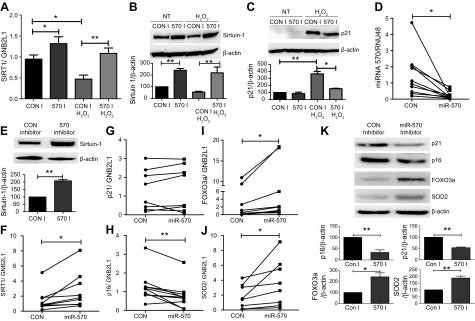
Inhibition of miR-570-3p rescues sirtuin-1 expression and modulates senescence markers. *A–C*) BEAS-2B cells (*n* = 7–10) treated with or without H_2_O_2_ and miRNA-570-3p antagomir (60 nM) or control and SIRT1 mRNA (*n* = 5) (*A*) and sirtuin-1 (*B*) and p21^Waf1^ (*C*) protein (*n* = 4) expression were examined. *D–F*) MiR-570-3p (*D*), sirtuin-1 protein (*E*), and SIRT1 mRNA (*F*) were detected in SAECs from patients with COPD treated with an miR-570-3p antagomir (60 nM) or control (*n* = 5–10). *G–J*) In these same cells, p21^Waf1^ (*G*), p16^INK4a^ (*H*), FOXO3a (*I*), and SOD2 (*J*) mRNA expression was detected. *K*) SAECs from patients with COPD treated with an miR-570-3p antagomir (60 nM) or control for 48 h and p21^Waf1^, p16^INK4a^, FOXO3a, and SOD2 protein expression were detected (*n* = 5). Data are means ± sem and were analyzed by using Kruskal-Wallis test with *post hoc* Dunn’s test, 1-way ANOVA with *post hoc* Bonferroni correction, unpaired or paired Student’s *t* test, and Wilcoxon signed rank test; *P < 0.05; **P < 0.01. GNB2L1, guanine nucleotide binding protein-polypeptide 2-like 1.

Previously, we and others have shown that oxidative stress–dependent down-regulation of sirtuin-1 in patients with COPD leads to loss of antioxidant gene expression and increased expression of MMP-9 (15, 17, 18, 20). Because miR-570-3p is elevated in patients with COPD and regulates sirtuin-1, we assessed whether this factor was a potential mechanism underlying sirtuin-1 reduction in COPD. Overexpression of an antagomir of miR-570-3p prevented oxidative stress–dependent reduction of sirtuin-1 at both the mRNA and protein levels (**Fig. 5*A, B***), while also reducing p21^Waf1^ induction by oxidative stress (Fig. 5*C*). We next confirmed miR-570-3p inhibition in SAECs from patients with COPD by overexpressing the antagomir (Fig. 5*D*) and found increased sirtuin-1 expression at both the mRNA and protein levels (Fig. 5*E, F*). In these same cells, p21^Waf1^ expression did not change, but a decrease in p16^INK4a^ mRNA expression was noted along with increased FOXO3a and SOD2 expression (Fig. 5*G–J*). Inhibition of miR-570-3p also reduced p27^kip1^ expression, while increasing CDK4 and human telomere reverse transcriptase (Supplemental Fig. S8). In addition to changes in gene expression, overexpression of the miR-570-3p antagomir decreased the protein levels of p21^Waf1^ and p16^INK4a^, while increasing the expression of FOXO3a and SOD2 (Fig. 5*K*). To determine whether miR-570-3p induced the expression of senescence markers *via* the direct down-regulation of sirtuin-1, an miR-570-3p antagomir was overexpressed and then sirtuin-1 silenced by using siRNA in BEAS-2B. In the presence of oxidative stress, silencing of sirtuin-1 after miR-570-3p inhibition failed to reduce p21, MMP-2, and IL-6 gene expression; the rescue of sirtuin-1 with the antagomir was also lost (Supplemental Fig. S9). Together, these data suggest that inhibition of miR-570-3p in COPD SAECs restores sirtuin-1 expression, leading to sirtuin-1–dependent inhibition of senescence markers and cellular rejuvenation.

### Inhibition of miR-570-3p induces cellular growth and suppresses SASP expression

**Figure 6:**
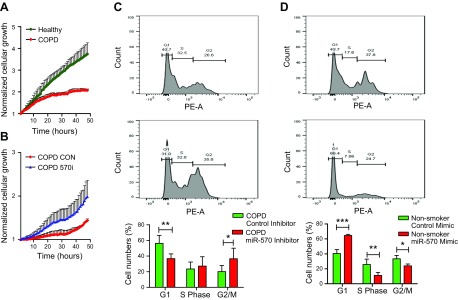
Inhibition of miRNA-570 expression in airway epithelial cells rescues cellular growth. *A*) Cellular proliferation measured by using the iCELLigence microelectronic biosensor system of SAECs from nonsmokers and patients with COPD (*n* = 4). *B*) Effect of miR-570-3p antagomir or control oligonucleotide on COPD SAECs proliferation (*n* = 4). *C*) Effect of miRNA-570-3p antagomir or oligonucleotide control, in COPD SAECs stained with propidium iodide and fluorescence detected by using flow cytometry on the PE-A channel (*n* = 5). *D*) Flow cytometric analysis of propidium iodide staining of miRNA-570-3p mimic or oligonucleotide control treated nonsmokers SAECs (*n* = 4). Data are means ± sem and were analyzed by using 2-way ANOVA with *post hoc* Bonferroni correction; *P < 0.05, **P < 0.01, ***P < 0.001.

**Figure 7:**
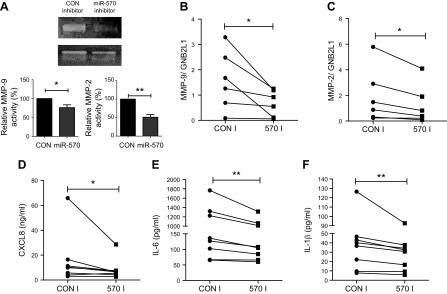
Inhibition of miRNA-570 expression in airway epithelial cells suppresses SASP release. *A*) Zymography of MMP-2 and MMP-9 expression measured in supernatants from COPD SAECs treated with miRNA-570-3p antagomir or oligonucleotide control (*n* = 5). *B*, *C*) MMP-9 (*B*) and MMP-2 (*C*) mRNA expression detected by qRT-PCR in SAECs after treatment with miRNA-570-3p antagomir or oligonucleotide control. *D–F*) Changes in CXCL8 (*D*), IL-6 (*E*), and IL-1β (*F*) release from COPD SAECs treated with either miRNA-570-3p antagomir or oligonucleotide control (*n* = 8). Data are means ± sem and were analyzed by using unpaired Student’s *t* test or Wilcoxon signed rank test; *P < 0.05, **P < 0.01.

Cell cycle arrest is a characteristic of senescent cells, with these cells ceasing to divide (8, 39). Utilizing iCELLigence technology for real-time monitoring of cellular growth, we observed that SAECs from patients with COPD had impaired cellular growth compared with nonsmoking, passage- and age-matched controls (**Fig. 6*A***). However, when transfected with an miR-570-3p antagomir, the growth of the COPD cells increased, suggesting miR-570-3p inhibition reversed cell cycle arrest (Fig. 6*B*). To confirm induced cellular growth, miR-570-3p was inhibited and cell cycle progression measured by using propidium iodide and flow cytometry. Inhibition of miR-570-3p appeared to induce progression through the cell cycle, with cells being driven out of G1 arrest (Fig. 6*C*). Conversely, SAEC from nonsmokers could be driven into G1 arrest by overexpressing miR-570-3p mimics (Fig. 6*D*). However, miR-570-3p inhibition in COPD SAECs significantly reduced the normally elevated levels of both protein and mRNA expression of MMP-2 and MMP-9 (**Fig. 7*A–C***), while inhibition of miR-570-3p in COPD SAECs also decreased CXCL8, IL-6, and IL-1β release (Fig. 7*D–F*). A trend toward increased release of CXCL8 and IL-1β in nonsmoker SAECs treated with the miR-570-3p mimic was also observed (Supplemental Fig. S10). These data suggest that inhibition of miR-570-3p is able to reverse stress-induced senescence by inhibiting cell cycle arrest and reducing the release of SASP proteins.

## DISCUSSION

Elevated numbers of senescent cells are found within the tissues of patients with age-associated diseases, implying the importance of cellular senescence in these conditions (40). Accelerated accumulation of senescent cells, caused by DNA damage and/or stress, may further exacerbate disease due to deregulated tissue repair *via* cell cycle arrest, as well as increasing levels of chronic inflammation *via* the SASP. These key features implicate dysfunctional cellular senescence as a key phenotype of accelerated aging diseases. Patients with COPD have elevated numbers of senescent cells within their lungs, with these being detected in many different cell types cultured *in vitro*, as well as in whole lung tissue samples (41–43). Here, we report a novel mechanism by which oxidative stress–driven p38-c-Jun signaling induces miR-570-3p expression. MiR-570-3p then directly binds to sirtuin-1, reducing its expression, leading to the induction of cellular senescence. We show that by inhibiting the elevated levels of miR-570-3p in SAECs from patients with COPD, we can increase cellular proliferation while reducing the expression of many key SASP proteins (MMP-2/9, IL-1β, CXCL8, and IL-6). These data suggest a key role for miR-570-3p in the induction of cellular senescence in COPD.

A comparison of the SASP released by senescent cells and the secretome of patients with COPD suggests that these processes are concordant, with the same pattern of cytokines, chemokines, and MMPs being released by senescent cells found in bronchoalveolar lavage fluid and sputum of patients with COPD (44, 45). Inhibition and/or clearance of these senescent cells may therefore reduce the proinflammatory milieu found within the lungs of patients with COPD and prevent disease progression. Our data suggest that by inhibiting miR-570-3p in COPD SAECs, we can reduce expression of MMP-2/9, IL-1β, IL-6, and CXCL8 (44, 46, 47). As the inhibition of sirtuin-1 induces MMP-9 expression *via* increased binding of acetylated NF-κB to the MMP-9 promoter (17), as well as regulating of CXCL8 and IL-6 expression *via* histone deacetylation (48), the down-regulation of sirtuin-1 by miR-570-3p may explain elevation of these proteins in COPD. These data imply that inhibition of miR-570-3p in COPD SAEC reduces expression of SASP-related proteins, thereby reducing the proinflammatory environment of the COPD lung.

Senescence of alveolar type II cells have been identified in patients with emphysema compared with nonsmokers (49). Senescence is proposed as a potential mechanism behind the destruction of the lung parenchyma surrounding the small airways, *via* reduced cellular proliferation and elevated proteases and cytokines of the SASP. In the present study, overexpressing the miR-570-3p antagomir restored cellular growth of COPD SAECs, occurring due to movement out of G1 cell cycle arrest, suggesting miR-570-3p inhibition can reverse one of the key phenotypes of cellular senescence. G1 cell cycle arrest may be induced by the activation of p53-p21 signaling (50). The acetylation of p53 induces subsequent expression of p21 (51), leading to the induction of cellular senescence. p53 is elevated in COPD lung homogenate samples compared with samples from non-COPD smoker controls and could be a potential mechanism behind elevated senescence in patients with COPD (52). p53 can be inhibited by deacetylation *via* sirtuin-1 (53). Therefore, the reduced expression of sirtuin-1 seen in patients with COPD, due in part to the elevated levels of miR-570-3p, may increase activation of p53 and be a potential mechanism by which cellular senescence is induced in these patients.

Oxidative stress is considered to be one of the major drivers underpinning the pathology of COPD (9), and it also induces cellular senescence (33, 54). We, along with others, have previously shown the importance of oxidative stress to induce miRNA and its importance in modulation of cellular senescence (20, 55, 56). The present study proposes a novel mechanism by which oxidative stress induces p38 signaling, leading to c-Jun–mediated transcription of miR-570-3p. Because p38 is activated in COPD cells, this mechanism may explain why elevated levels of miR-570-3p are found in lung homogenate samples of patients with COPD (34). Previous studies have linked miR-570 expression and exposure of BEAS-2B cells to particulate matter with an aerodynamic diameter <2.5 μm. In these experiments, particulate matter with an aerodynamic diameter <2.5 μm led to hypomethylation of the transcription start site of miR-570, leading to increased expression of this miRNA species (57). These data suggest that elevated oxidative stress through cigarette smoke exposure and/or air pollution may also induce miR-570-3p in airway epithelial cells, inducing cellular senescence.

In summary, we show for the first time that miR-570-3p is elevated in COPD and drives a senescent phenotype in airway epithelial cells. MiR-570-3p is induced by oxidative stress *via* p38 MAPK-dependent AP-1–mediated transcription, leading to down-regulation of sirtuin-1. Up-regulation of miR-570-3p induces cycle arrest and SASP proteins, with increased proinflammatory cytokine and MMP expression. As the clearance of senescent cells prolongs the life span of mammals (58), our current research suggests that preventing cellular senescence may decrease the accelerated ageing phenotype in COPD and potential associated multimorbidities *via* targeting miR-570-3p therapeutically.

## Supplementary Material

This article includes supplemental data. Please visit *http://www.fasebj.org* to obtain this information.

## Abbreviations

CDK4: cyclin-dependent kinase 4
COPD: chronic obstructive pulmonary disease
CXCL8: C-X-C motif chemokine ligand 8
FOXO: forkhead box class O
H_2_O_2_: hydrogen peroxide
miRNA: micro-RNA
MMP: matrix metalloproteinase
SAEC: small airway epithelial cell
SASP: senescence-associated secretory phenotype
SOD2: superoxide dismutase 2
