# Supplementary material for: MicroRNA-570 is a novel regulator of cellular senescence and inflammaging
Source: FASEB J. 2018 Aug 29;33(2):1605–16. doi: 10.1096/fj.201800965R (PMC6338629; doi:10.1096/fj.201800965R)
Supplement: Supplementary file 1 [file fj.201800965R.sf1.pdf]

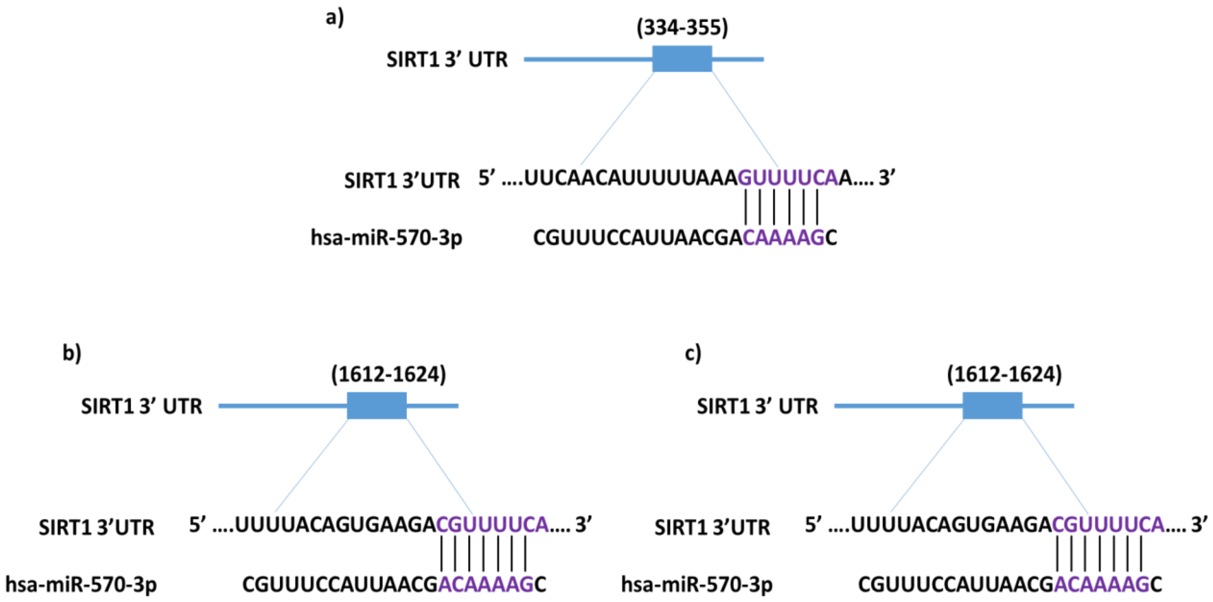

Supplementary Fig. 1. Putative miR-570-3p binding sites in the 3'UTR of SIRT1

3 putative binding sites of where miR-570-3p binds to the 3'UTR of sirtuin-1 gene identified using bioinformatics software which predict miRNA binding sites.
