# Supplementary material for: MicroRNA-570 is a novel regulator of cellular senescence and inflammaging
Source: FASEB J. 2018 Aug 29;33(2):1605–16. doi: 10.1096/fj.201800965R (PMC6338629; doi:10.1096/fj.201800965R)
Supplement: Supplementary file 2 [file fj.201800965R.sf2.pdf]

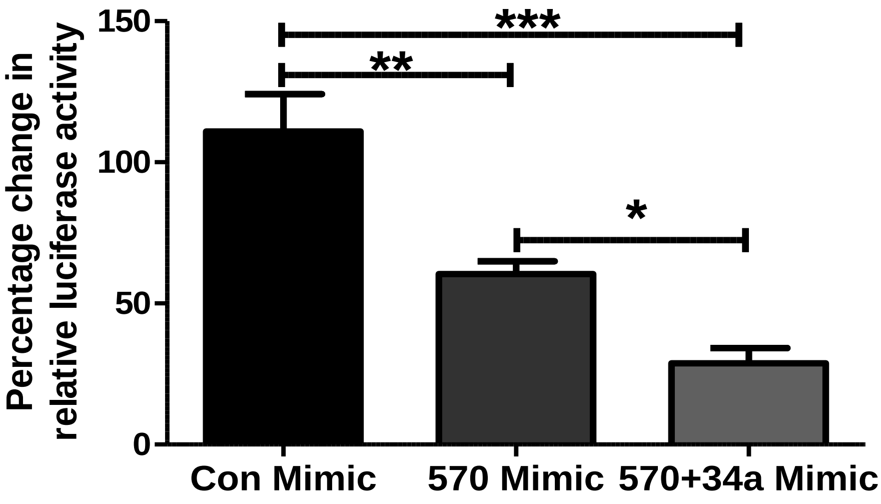

Supplementary Fig. 2. miR-570-3p acts independently to miR-34a

BEAS2Bs were co-transfected with a control and SIRT1 luciferase reporter, along with either a miR-570-3p mimic +/- a miR-34a mimic or mimic control for 24 hours and relative luciferase activity detected. Data are means ± SEM and analyzed by One way Anova post hoc Bonferroni. * P ≤ 0.05, **P ≤ 0.01, ***P ≤ 0.001
