# Supplementary material for: MicroRNA-570 is a novel regulator of cellular senescence and inflammaging
Source: FASEB J. 2018 Aug 29;33(2):1605–16. doi: 10.1096/fj.201800965R (PMC6338629; doi:10.1096/fj.201800965R)
Supplement: Supplementary file 3 [file fj.201800965R.sf3.pdf]

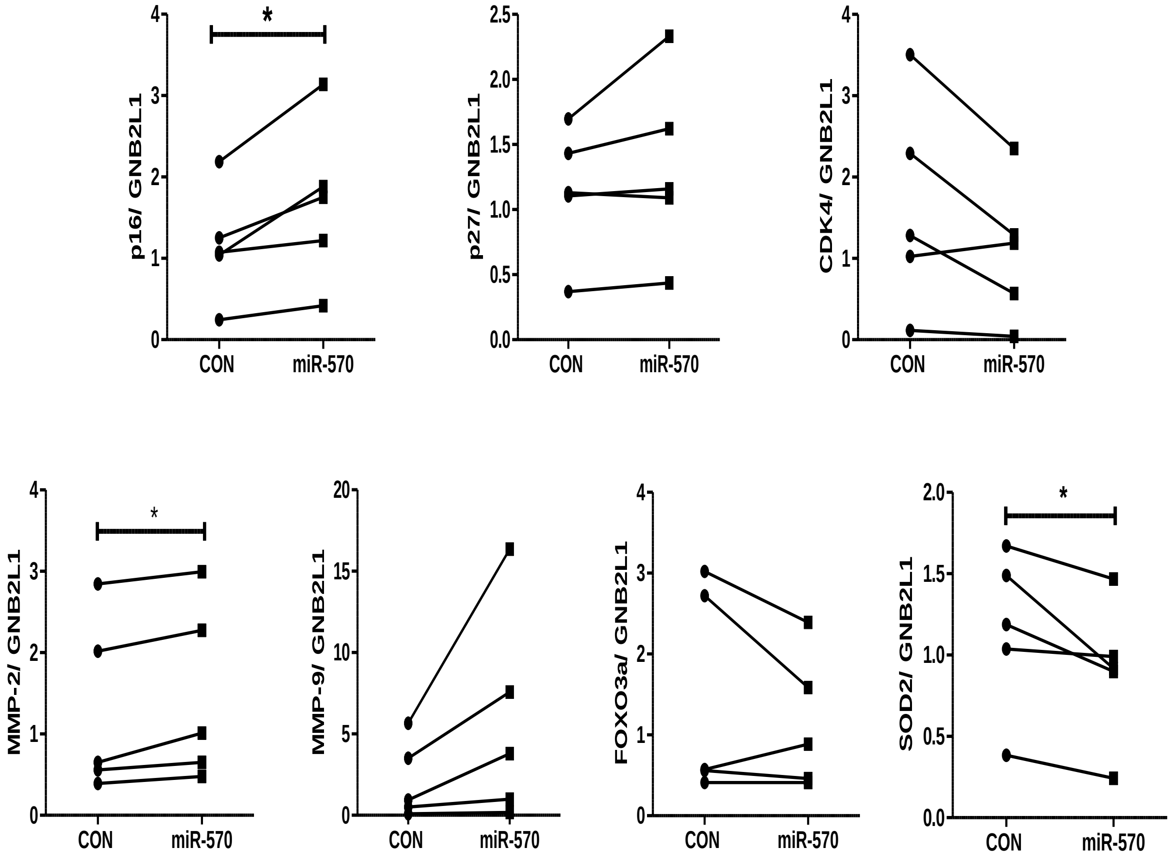

Supplementary Fig. 3. miR-570-3p mimics modulate cell cycle checkpoint inhibitors, SASP and antioxidant gene expression

SAECs from 5 non-smokers were treated with miR-570-3p mimics for 48 hours. RNA was extracted and changes in gene expression of p16 ^{INK4a}, p27 ^{Kip1}, CDK4, MMP-2, MMP-9, SOD2 and FOXO3a were assessed by qRT-PCR using a TaqMan assays normalized to GNB2L1. Data are analyzed Wilcoxon matched-pairs signed rank test. * P ≤ 0.05, **P ≤ 0.01, ***P ≤ 0.001
