# Supplementary material for: MicroRNA-570 is a novel regulator of cellular senescence and inflammaging
Source: FASEB J. 2018 Aug 29;33(2):1605–16. doi: 10.1096/fj.201800965R (PMC6338629; doi:10.1096/fj.201800965R)
Supplement: Supplementary file 4 [file fj.201800965R.sf4.pdf]

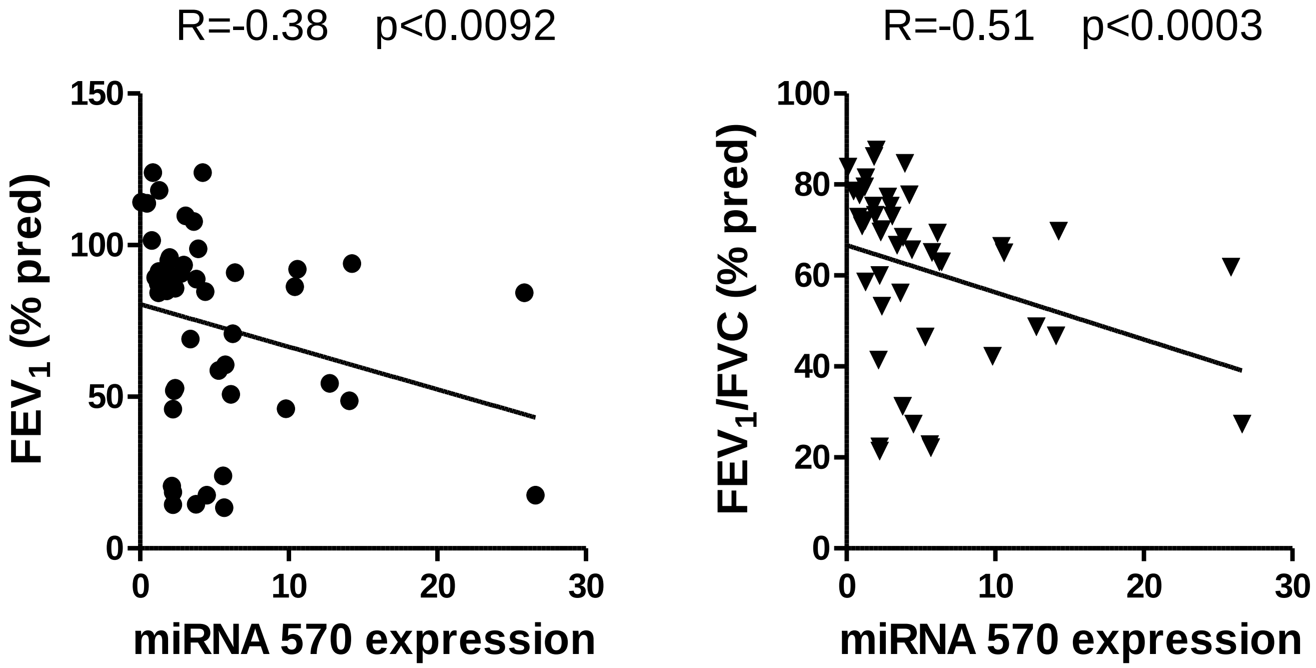

Supplementary Fig. 4. Correlation between disease severity and miR-570-3p expression

Correlation data between miR-570-3p expression in lung homogenate samples and FEV_1 (% predicted) and FEV_1/FVC (% predicted). Data was analyzed by Spearman correlation test.
