# Supplementary material for: MicroRNA-570 is a novel regulator of cellular senescence and inflammaging
Source: FASEB J. 2018 Aug 29;33(2):1605–16. doi: 10.1096/fj.201800965R (PMC6338629; doi:10.1096/fj.201800965R)
Supplement: Supplementary file 5 [file fj.201800965R.sf5.pdf]

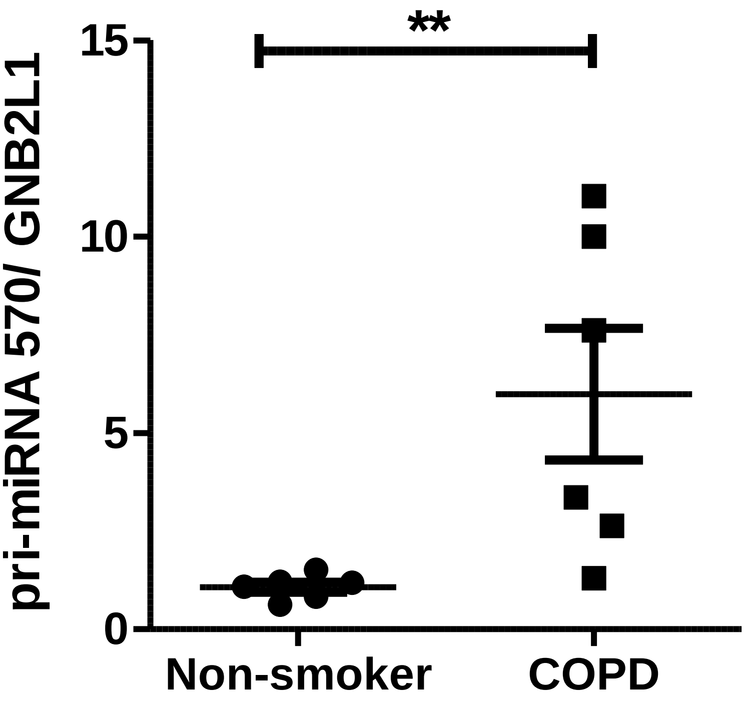

Supplementary Fig. 5. Expression of pri-miR-570-3p in COPD SAEC compared to non-smoking controls

Pri-M-570-3p expression was detected in SAEC from non-smokers (n=6) and COPD (n=6) subjects. Data are analyzed by Mann-Whitney U test. **P ≤ 0.01
