# Supplementary material for: MicroRNA-570 is a novel regulator of cellular senescence and inflammaging
Source: FASEB J. 2018 Aug 29;33(2):1605–16. doi: 10.1096/fj.201800965R (PMC6338629; doi:10.1096/fj.201800965R)
Supplement: Supplementary file 6 [file fj.201800965R.sf6.pdf]

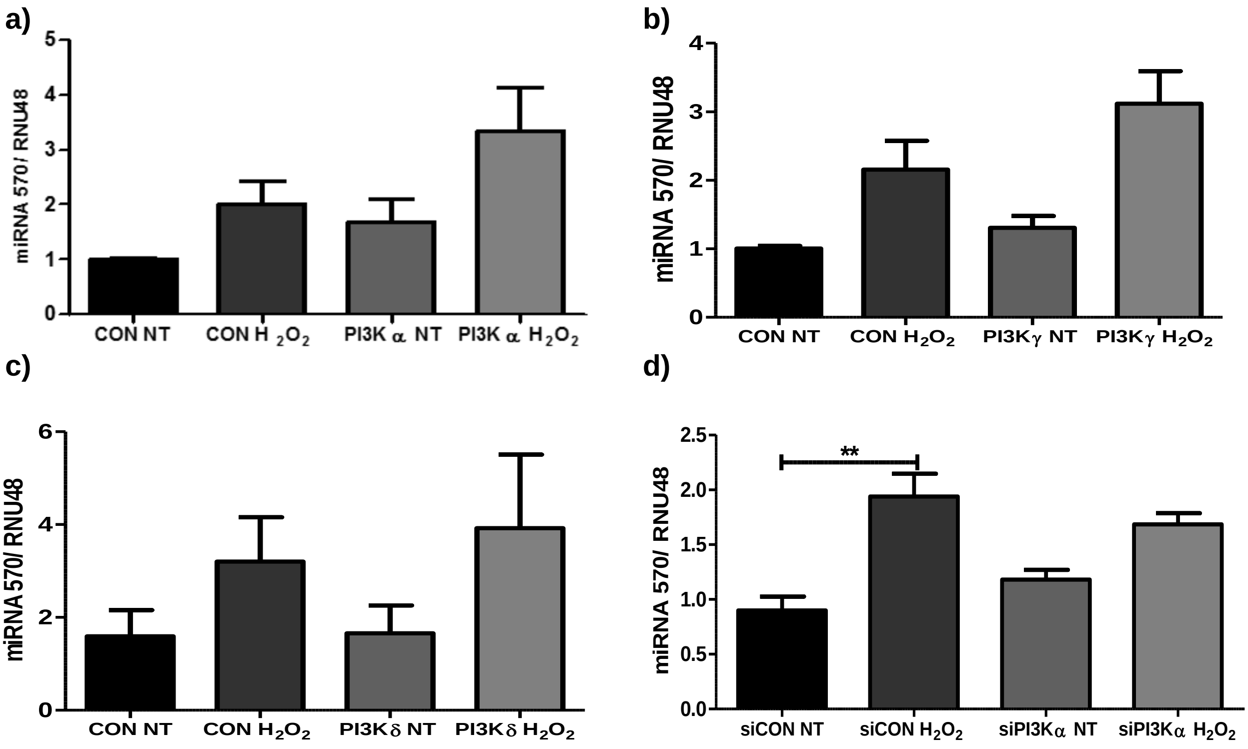

Supplementary Fig. 6. PI3K signalling does not regulate miR-570-3p expression

(A-C) BEAS-2B cells were treated with PIK75, AS-605240 or IC-87114 (10 µM) or vehicle (DMSO) for 1 hour prior to stimulation with or without 100 µM H_2O_2 for 48 hours, RNA extracted and miR-570-3p levels assessed (N=3-5). (D) BEAS-2B cells were treated with either random oligonucleotide controls or siPI3Kα for 24 hours prior to stimulation with or without 100 µM H_2O_2 for 48 hours, RNA extracted and miR-570-3p levels assessed (N=4). Data are means ± SEM and analysed by Kruskal-Wallis test with Dunn's Multiple Comparison Test. *P ≤ 0.05
