# Supplementary material for: MicroRNA-570 is a novel regulator of cellular senescence and inflammaging
Source: FASEB J. 2018 Aug 29;33(2):1605–16. doi: 10.1096/fj.201800965R (PMC6338629; doi:10.1096/fj.201800965R)
Supplement: Supplementary file 7 [file fj.201800965R.sf7.pdf]

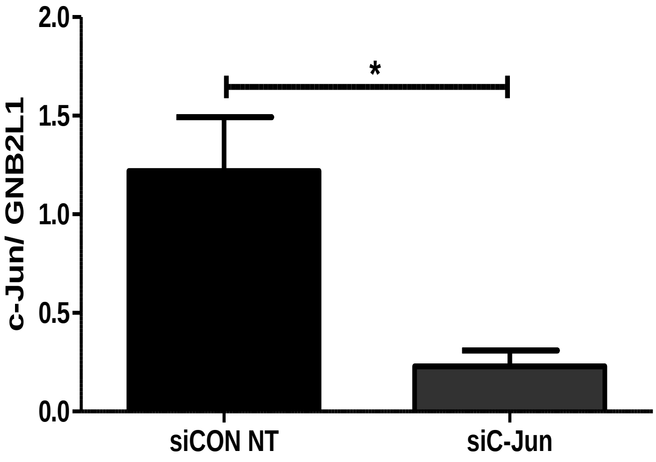

Supplementary Fig. 7. Silencing of c-Jun

BEAS-2B cells were transfected with small interfering RNA (siRNA) against either c-Jun for 48h or a random oligonucleotide control and c-Jun gene expression examined (N=5). Data are means ± SEM and analyzed by Mann-Whitney U test. * $P \leq 0.05$.
