# Supplementary material for: MicroRNA-570 is a novel regulator of cellular senescence and inflammaging
Source: FASEB J. 2018 Aug 29;33(2):1605–16. doi: 10.1096/fj.201800965R (PMC6338629; doi:10.1096/fj.201800965R)
Supplement: Supplementary file 8 [file fj.201800965R.sf8.pdf]

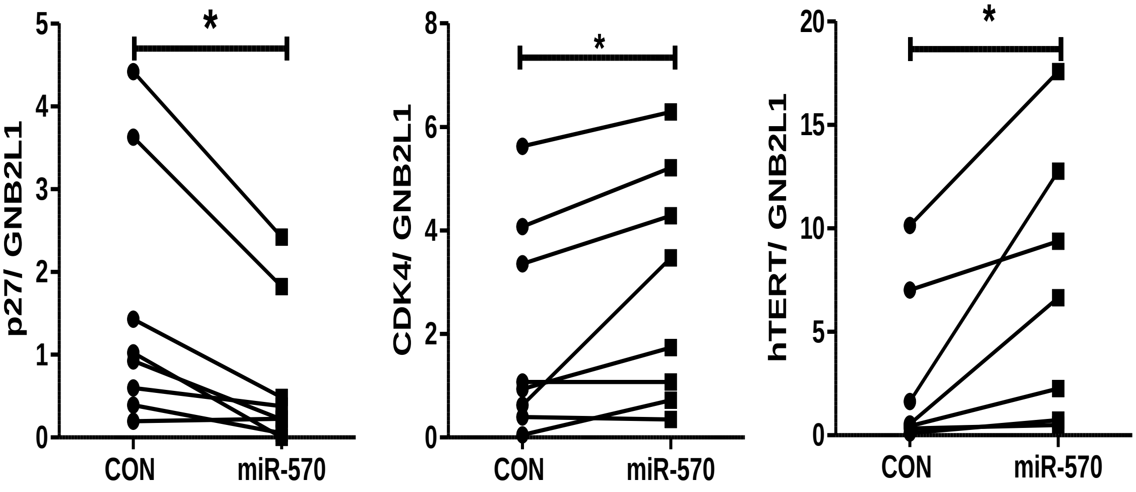

Supplementary Fig. 8. Effect of miR-570-3p antagomir in COPD SAEC on mRNA gene expression

COPD SAEC were transfected with a miR-570-3p antagomir or random oligonucleotide control and p27, CDK4 and hTERT gene expression examined (N=5). Data are means ± SEM and analysed by Wilcoxon matched-pairs signed rank test. * P ≤ 0.05.
