# Supplementary material for: MicroRNA-570 is a novel regulator of cellular senescence and inflammaging
Source: FASEB J. 2018 Aug 29;33(2):1605–16. doi: 10.1096/fj.201800965R (PMC6338629; doi:10.1096/fj.201800965R)
Supplement: Supplementary file 9 [file fj.201800965R.sf9.pdf]

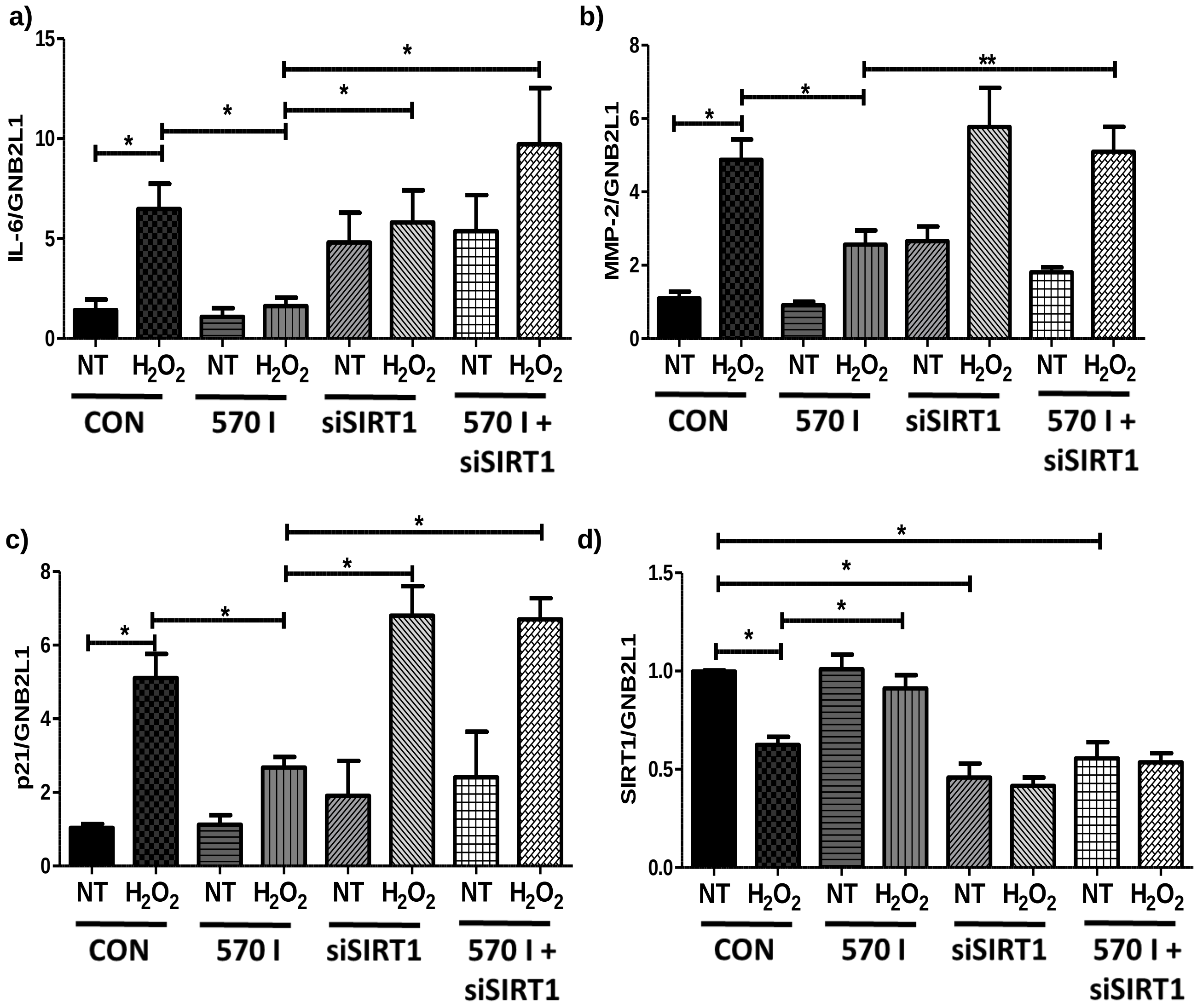

Supplementary Fig. 9. Effect of miR-570-3p antagomir in COPD SAEC on mRNA gene expression

BEAS-2B cells were transfected with a miR-570-3p antagomir or random oligonucleotide control for 24 hours and then transfected with either siRNA control or SIRT1 siRNA for 24 hours. Cells were then serum starved before being treated with H_2O_2 for 48 hours. RNA was then extracted and RT-qPCR performed for a) IL-6 b) MMP-2, c) p21 and d) SIRT1 gene expression examined (N=5). Data are means ± SEM and analysed by Kruskal-Wallis test with Dunn's Multiple Comparison Test. * $P \leq 0.05$.
