# Supplementary material for: MicroRNA-570 is a novel regulator of cellular senescence and inflammaging
Source: FASEB J. 2018 Aug 29;33(2):1605–16. doi: 10.1096/fj.201800965R (PMC6338629; doi:10.1096/fj.201800965R)
Supplement: Supplementary file 10 [file fj.201800965R.sf10.pdf]

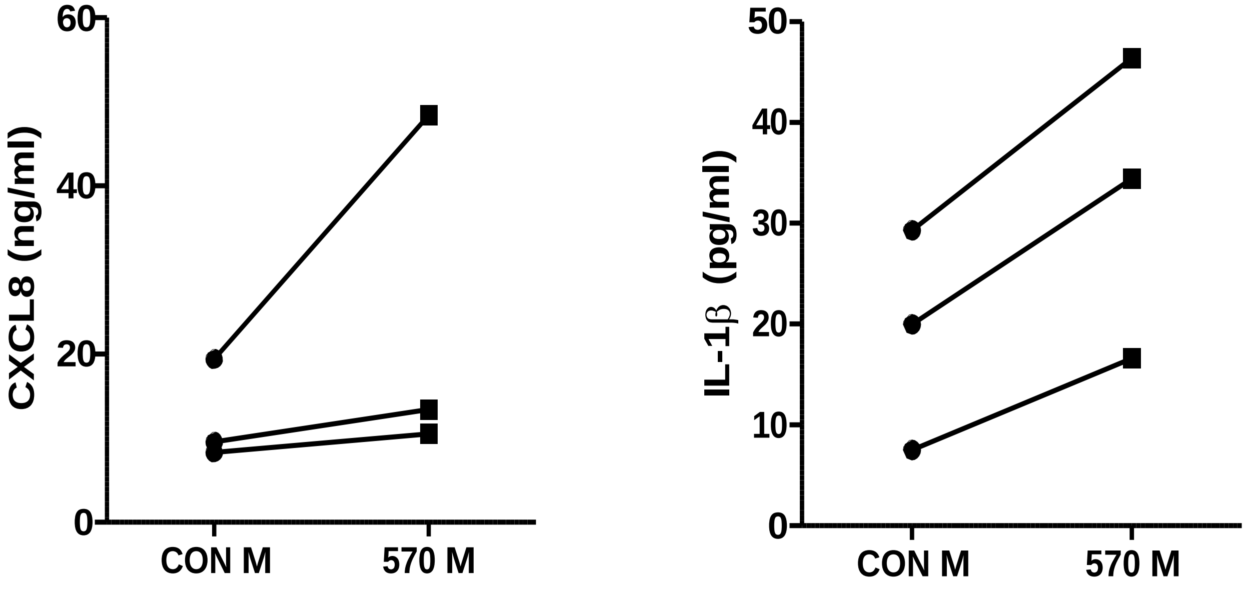

Supplementary Fig. 10. Over-expression of miR-570-3p induces cytokine release SAECs from 3 non-smokers were treated with miR-570-3p mimics for 48 hours and supernatant collected, changes in CXCL-8 and IL-1β were detected by ELISA.
